# Supplementary material for: From sequence to enzyme mechanism using multi-label machine learning
Source: BMC Bioinformatics. 2014 May 19;15:150. doi: 10.1186/1471-2105-15-150 (PMC4229970; doi:10.1186/1471-2105-15-150)
Supplement: Additional file 2 — Java code of ml2db. Additional file ml2db_code.tar.gz contains the Java source code to run the multi-label machine learning experiments and save the results to database. The code’s Javadoc is included. [file 1471-2105-15-150-S2.zip › additional file 2/ml2db/utils/doc/uk/ac/standrews/utils/main/database/class-use/Table.html]

Uses of Class uk.ac.standrews.utils.main.database.Table


JavaScript is disabled on your browser.


- Overview
- Package
- Class
- Use
- Tree
- Deprecated
- Index
- Help

- Prev
- Next

- Frames
- No Frames

- All Classes

## Uses of Class uk.ac.standrews.utils.main.database.Table

- Packages that use Table

  | Package | Description |
  | --- | --- |
  |  |  |
  | --- | --- |
  | uk.ac.standrews.utils.main.database |  |
  | uk.ac.standrews.utils.test.database |  |
- - ### Uses of Table in uk.ac.standrews.utils.main.database

    Methods in uk.ac.standrews.utils.main.database that return Table

    | Modifier and Type | Method and Description |
    | --- | --- |
    |  |  |
    | --- | --- |
    | `Table` | TableManager.`getTable()` |

    Constructors in uk.ac.standrews.utils.main.database with parameters of type Table

    Constructor and Description

    `TableCreator(Table table)`

    `TableManager(Table table)`

    Constructor to create a manager to create, read and write a table.

    `TableReader(Table table)`

    `TableRow(Table table)`

    `TableRow(Table table,
    java.util.TreeMap<java.lang.String,java.lang.String> columnToValueMap)`

    `TableRow(Table table,
    java.util.Vector<java.lang.String> columns,
    java.util.Vector<java.lang.String> values)`

    `TableWriter(Table table)`

    Constructor 2: takes the table name and a list of fields
  - ### Uses of Table in uk.ac.standrews.utils.test.database

    Methods in uk.ac.standrews.utils.test.database that return Table

    | Modifier and Type | Method and Description |
    | --- | --- |
    |  |  |
    | --- | --- |
    | `static Table` | TableTest.`getTest1Table()` |

- Overview
- Package
- Class
- Use
- Tree
- Deprecated
- Index
- Help

- Prev
- Next

- Frames
- No Frames

- All Classes
